# Time-resolved Rayleigh scattering measurements of methane clusters for laser-cluster fusion experiments

**DOI:** 10.1371/journal.pone.0261574

**Published:** 2021-12-17

**Authors:** J. Song, J. Won, W. Bang

**Affiliations:** 1 Department of Physics and Photon Science, GIST, Gwangju, South Korea; 2 Center for Relativistic Laser Science, Institute for Basic Science, Gwangju, South Korea; Rutgers University Newark, UNITED STATES

## Abstract

We present a time-resolved analysis of Rayleigh scattering measurements to determine the average size of methane clusters and find the optimum timing for laser-cluster fusion experiments. We measure Rayleigh scattering and determine the average size of methane clusters varying the backing pressure (*P*_0_) from 11 bar to 69 bar. Regarding the onset of clustering, we estimate that the average size of methane clusters at the onset of clustering is *N*_c0_≅20 at 11 bar. According to our measurements, the average cluster radius *r* follows the power law of *r*∝*P*_0_^1.86^. Our ion time-of-flight measurements indicate that we have produced energetic deuterium ions with *kT* = 52±2 keV after laser-cluster interaction using CD_4_ gas at 50 bar. We find that this ion temperature agrees with the predicted temperature from CD_4_ clusters at 50 bar with *r* = 14 nm assuming the Coulomb explosion model.

## Introduction

The use of deuterated clusters has gathered some interest in fusion science using high-power lasers for the past two decades [1–9]. In a laser-cluster-fusion experiment, the laser-matter interaction leads to an explosion of individual clusters generating energetic deuterium ions. The so-called Coulomb explosion model has been fairly successful in explaining how and how much the ions gain kinetic energy from the incident laser pulse [2, 10, 11]. The kinetic energy of deuterium ions after Coulomb explosion is often sufficiently high to produce nuclear fusion reactions [1–9]. Assuming that deuterated clusters are fully-ionized and the entire Coulomb potential is converted to the kinetic energy of exploding ions, the ion temperature of deuterium fusion plasmas increases with the average size of clusters [2, 11].

Rayleigh scattering is one of the commonly used methods for cluster size measurements. The average size of clusters is calculated using the ratio of gas temperatures, backing pressures, and scattering signal intensities. Since this method can only tell us the relative cluster sizes [12], it requires information about the gas jet conditions at the onset of clustering. The average size of clusters at the onset of clustering is often measured from mass spectrometries [13] and other techniques [12] for atomic or molecular clusters. In this paper, we have estimated this by comparing Rayleigh scattering cross-sections, and determined the average sizes of methane clusters at backing pressures varying from 11 bar to 69 bar.

In the Coulomb explosion model, the ion temperature, which we approximate as two-thirds of the average ion kinetic energy, can be calculated from the measured average cluster size [8, 11]. We compare the ion temperatures calculated from the measured cluster sizes with the ion temperatures determined from the ion time-of-flight measurements. Our experimental results show that these two ion temperatures agree with each other, and we confirm the validity of size measurements of methane clusters using Rayleigh scattering.

Furthermore, we investigate the optimum timing for laser-cluster fusion reactions using time-resolved Rayleigh scattering analysis. Time-averaged scattering signals have shown a maximum signal intensity indicating either the largest average cluster size or the most dense clusters inside the scattering volume, both of which enhance the fusion yields.

## Materials and methods

### Experimental setup

**Fig 1:**
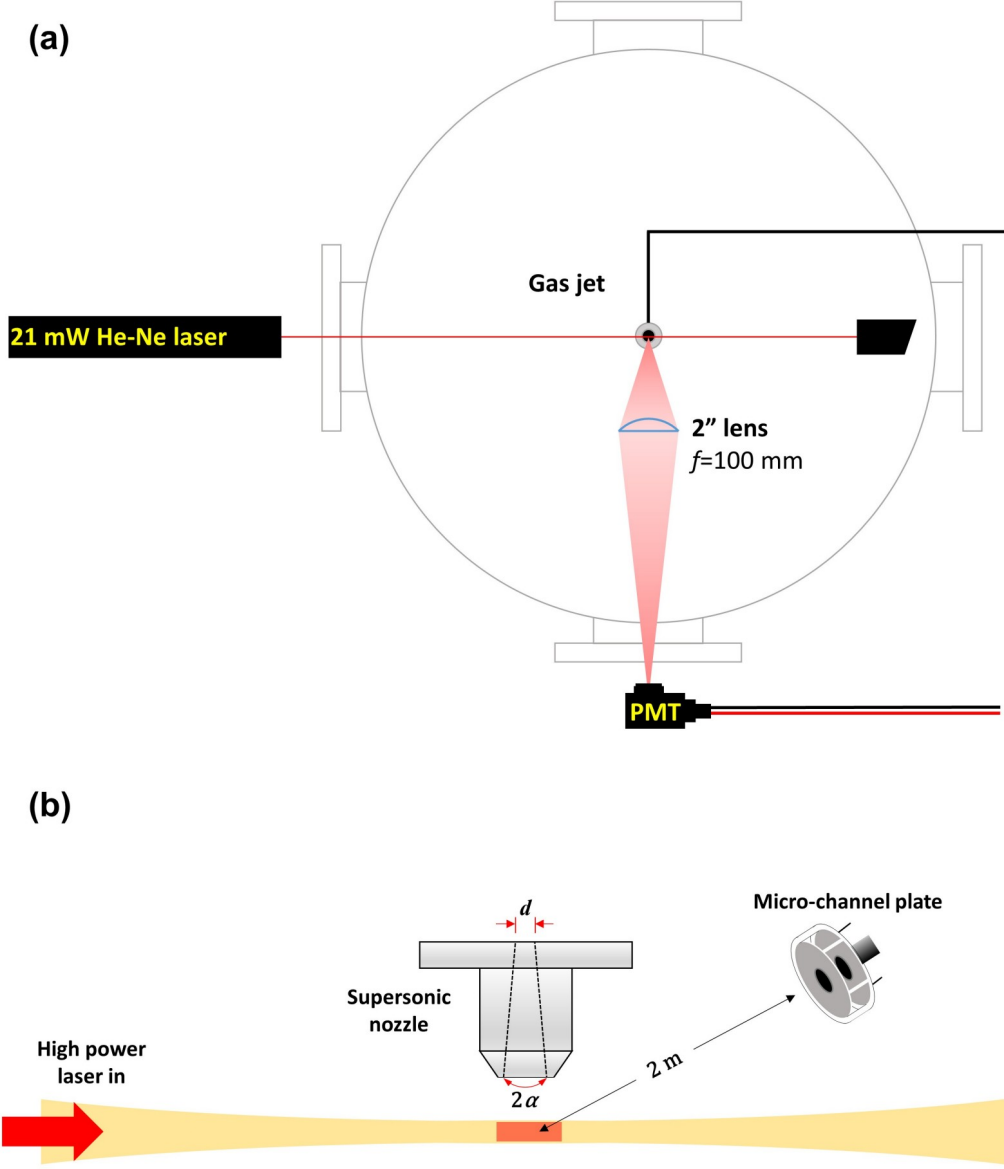
Schematic layout of the experimental setup. Experimental setups for (a) Rayleigh scattering measurements and (b) ion time-of-flight measurements from laser-cluster fusion plasmas. The same nozzle was used for both measurements with an orifice diameter of *d* = 0.79 mm and an expansion half-angle of *α* = 5°.

The experiments were carried out to measure the average size of the deuterated methane (CD_4_) clusters for laser-cluster fusion experiments. For the Rayleigh scattering measurements, we used methane gas (CH_4_) instead of deuterated methane (CD_4_) owing to its much lower cost. Fig 1(A) shows the schematic diagram of our Rayleigh scattering experiments. A 21 mW continuous wave He-Ne laser with a wavelength of 632.8 nm and a beam diameter of 0.7 mm was used to produce Rayleigh scattered light. The gas jet inside a vacuum target chamber was regulated by a solenoid pulse valve (Parker Series 9) with an opening time of 1 ms, operated by Parker IOTA ONE valve driver. Methane clusters were formed from the supersonic expansion at room temperature through a conical nozzle with an orifice diameter of 0.79 mm and an expansion half-angle of 5°. The Rayleigh scattered light was collected using a 2-inch AR coated plano-convex lens with a focal length of 100 mm, then the signal was amplified by Hamamatsu R928 photo-multiplier tube (PMT). A 1 GHz oscilloscope recorded the amplified scattering signal as a function of time. Fig 1(B) shows the experimental setup for the temperature measurements of cluster fusion plasmas. For the laser-cluster fusion experiments, we used a 100 TW Ti:Sapphire laser system at the Center for Relativistic Laser Science (CoReLS). An f/40 spherical mirror focused 3 J, 30 fs laser pulses (at 800 nm wavelength) onto CD_4_ clusters at an intensity as high as ~1×10^18^ Wcm^-2^, and produced energetic deuterium ions suitable for the cluster-fusion experiments. These energetic ions were detected by a micro-channel plate (MCP), Hamamatsu F9890-13, which was installed 2 m away from the target chamber center. The MCP recorded the ion time-of-flight signals, and measured the deuterium ion temperatures experimentally.

### Time-resolved Rayleigh scattering measurements

Since the timescale of the valve opening (~1 ms) is significantly longer than the laser pulse duration (25–30 fs) for laser-cluster fusion experiments, we expect there could be an optimum timing for the fusion reactions. Typical Rayleigh scattering measurements using imaging systems [6] lose their time-resolved information while integrating the signals over time. For example, the pixel value in a CCD camera corresponds to the total accumulated number of scattered photons during the whole exposure time, losing all the time information. A PMT connected with a high impedance oscilloscope can show some time history from the scattering events, but the resolution is usually poor and cannot show individual photon counts. We have used an impedance-matched (50 Ω) oscilloscope with a PMT for a much better temporal resolution (~ns). We estimate the time resolution of our detection system to be few nanoseconds based on the 2.2 ns rise time of R928 PMT.

**Fig 2:**
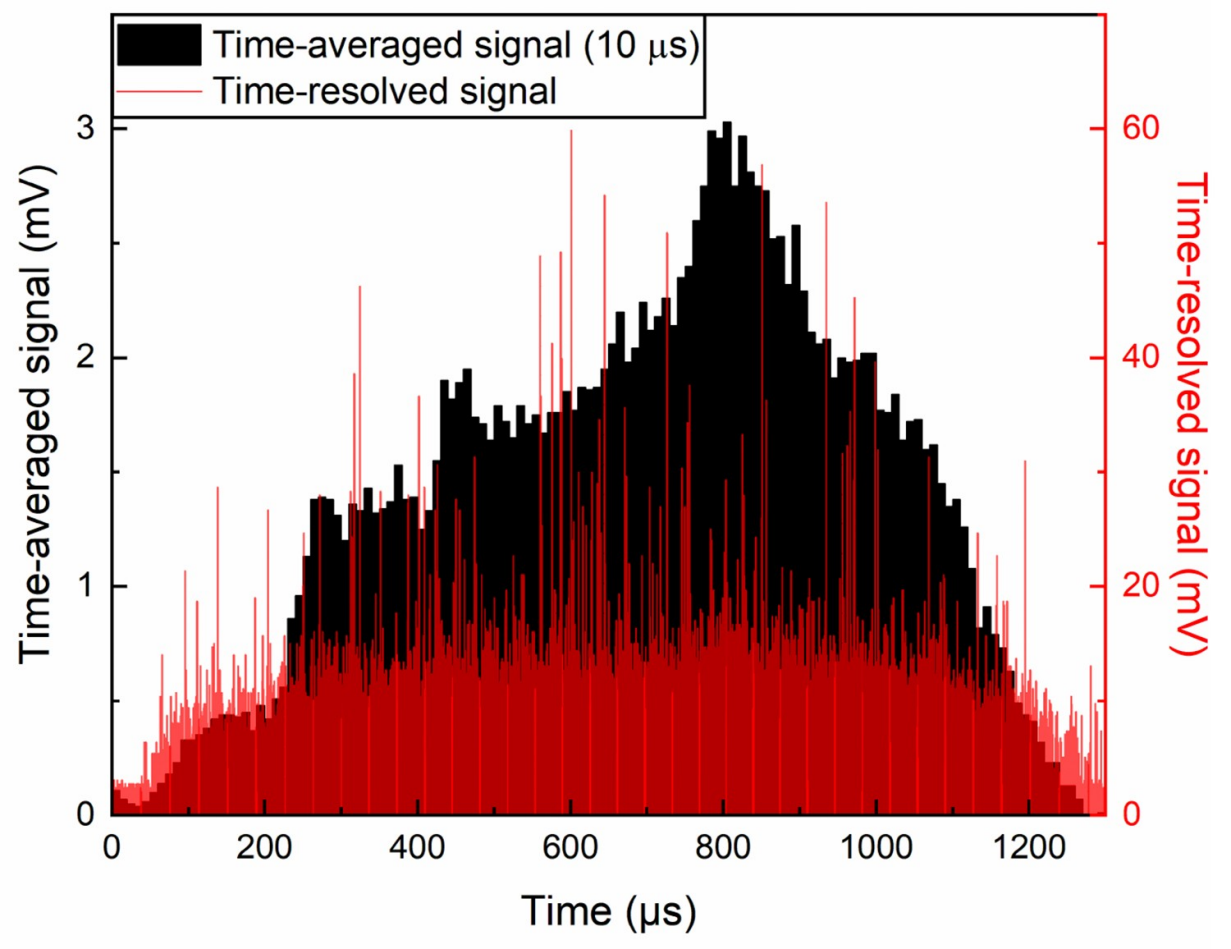
Time-averaged and time-resolved Rayleigh scattering signals. The red lines indicate time-resolved Rayleigh scattering signals from methane clusters produced at a backing pressure of 20 bar with a 1 ms valve opening time. The black bars indicate time-averaged signals during 10 μs intervals. The time-averaged signal reaches its peak around 800 μs with a full width at half maximum of ~700 μs.

Fig 2 shows the time-resolved (red lines) and time-averaged (black bars) Rayleigh scattering signals at a backing pressure of 20 bar. The valve starts opening at time zero and starts closing after 1 ms. The time-resolved scattering signals present numerous peaks, some of which are large, corresponding to multiple photons arriving at the same time. A single large peak, however, does not directly translate to a large cluster owing to the statistical nature of this type of measurements. Instead, we need to consider the total number of photons arriving at the detector within a finite time interval. Therefore, we have divided the 1 ms opening time into ~100 intervals, averaging the scattered signals every 10 μs.

The time-averaged signals in Fig 2 show a distinct trend of the scattered signal intensity, contrary to the time-resolved signals. We can interpret a higher signal intensity originating either from a larger cluster size or from a larger number of clusters. Both conditions are desirable for laser-cluster fusion experiments since a larger cluster is known to produce more energetic ions after Coulomb explosion and a large number of clusters means more targets. The black bars in Fig 2 show a maximum time-averaged signal at around 800 μs after the valve opening, which hints the existence of an optimum timing for laser-cluster interactions. Although several large peaks (red lines) are present at around 600 μs in Fig 2, we find that there are more signals at around 800 μs on a closer look (See Fig A in S1 File). We present a quantitative analysis regarding this matter in section S1.1 of S1 File.

Using measured molecular number density (10^17^–10^18^ molecules/cm^3^) of methane cluster jet in previous experiments [3, 6] and the average cluster size measured in our experiments, we expect the average time between Rayleigh scattering signals to be 8–80 ns at a backing pressure of 20 bar. This suggests that our detection time resolution is sufficient for time-resolved scattering measurements (see Fig B in S1 File). In S1.3 of S1 File, we show how we calculate the average time between Rayleigh scattering signals for a molecular number density of ~10^17^ molecules/cm^3^.

### Cluster size calculations

In our measurements, we assume the onset of clustering occurs when the Rayleigh scattering signal exceeds twice the background noise level. Previous studies suggest that the number of atoms at the onset of clustering is *N*_c0_≅100 for atomic deuterium clusters [14, 15]. In the case of methane, the cluster size which scatters a similar amount of light as a deuterium cluster with 100 atoms can be estimated by comparing the differential cross-sections [12, 16], dσ/dΩ,

$$\frac{d\sigma }{d\Omega }(90{}^{\circ})=\frac{{16\pi }^{4}r^{6}}{\lambda^{4}}{\left(\frac{n_{i}^{2}-1}{n_{i}^{2}+2}\right)}^{2},$$ (1)

where *λ* = 632.8 nm is the wavelength of the incident He-Ne laser beam, *r* is the radius of a deuterium or a methane cluster, and *n*_*i*_ is the refractive index of the corresponding cluster. The refractive indices of liquid deuterium and liquid methane are approximately 1.13 [17, 18] and 1.27 [19], respectively. Therefore, Eq (1) implies that a methane cluster whose radius is 80% that of a deuterium cluster scatters a similar amount of light.

Using the known number density of liquid deuterium (*n*_D_ = 4.86×10^22^ atoms/cm^3^) and liquid methane (*n*_CH4_ = 1.85×10^22^ molecules/cm^3^), we estimate the number of molecules inside a methane cluster at the onset of clustering as *N*_c0_≅20. The scattering cross-section of a methane cluster composed of 20 molecules is comparable to that of a deuterium cluster with 100 atoms, and the methane cluster will scatter a similar amount of light.

Since *r*∝*N*_c_^1/3^, the Rayleigh scattering intensity, *I*_RS_, can be written as

$$I_{RS}\propto n_{c}\frac{d\sigma }{d\Omega }\propto n_{c}N_{c}^{2},$$ (2)

where *n*_*c*_ is the number density of clusters, and *N*_*c*_ is the average number of molecules inside each cluster. Meanwhile, the number density of methane molecules, *n*_CH4_, at the nozzle output can be expressed using *n*_*c*_ and *N*_*c*_ as *n*_*CH*4_ = *n_c_N_c_*, and it is known to be proportional to the number density of the input gas before the nozzle [20]. Therefore, *I*_RS_ can then be expressed as [21]

$$I_{RS}\propto n_{CH4}N_{c}\propto \frac{P_{0}}{T_{0}}N_{c},$$ (3)

where *P*_0_ is the backing pressure, and *T*_0_ is the temperature of methane. Rayleigh scattering measurements using the same nozzle geometry but with different gas jet conditions share the same proportionality constant, *k*, given by

$$I_{RS}=k\frac{P_{0}}{T_{0}}N_{c},\;I_{RS}^{\prime }=k\frac{P_{0}^{\prime }}{T_{0}^{\prime }}N_{c}^{\prime }.$$ (4)

Taking the second relation in Eq (4) as the onset values, the average size of methane clusters is expressed as follows

$$N_{c}=\frac{I_{RS}}{I_{RS}^{onset}}\frac{P_{0}^{onset}}{P_{0}}\frac{T_{0}}{T_{0}^{onset}}N_{c0}.$$ (5)

Using *r* = 0.234×*N*_c_^1/3^ (nm) [11], the average radius of methane clusters is given by

$$r[nm]=0.234\times {\left(N_{c0}\times \frac{I_{RS}}{I_{RS}^{onset}}\frac{P_{0}^{onset}}{P_{0}}\frac{T_{0}}{T_{0}^{onset}}\right)}^{1/3},$$ (6)

where *N*_c0_≅20, $P_{0}^{onset}$≅11 bar, and $T_{0}(=T_{0}^{onset})$ = 293 K in our experiments.

## Results and discussion

**Fig 3:**
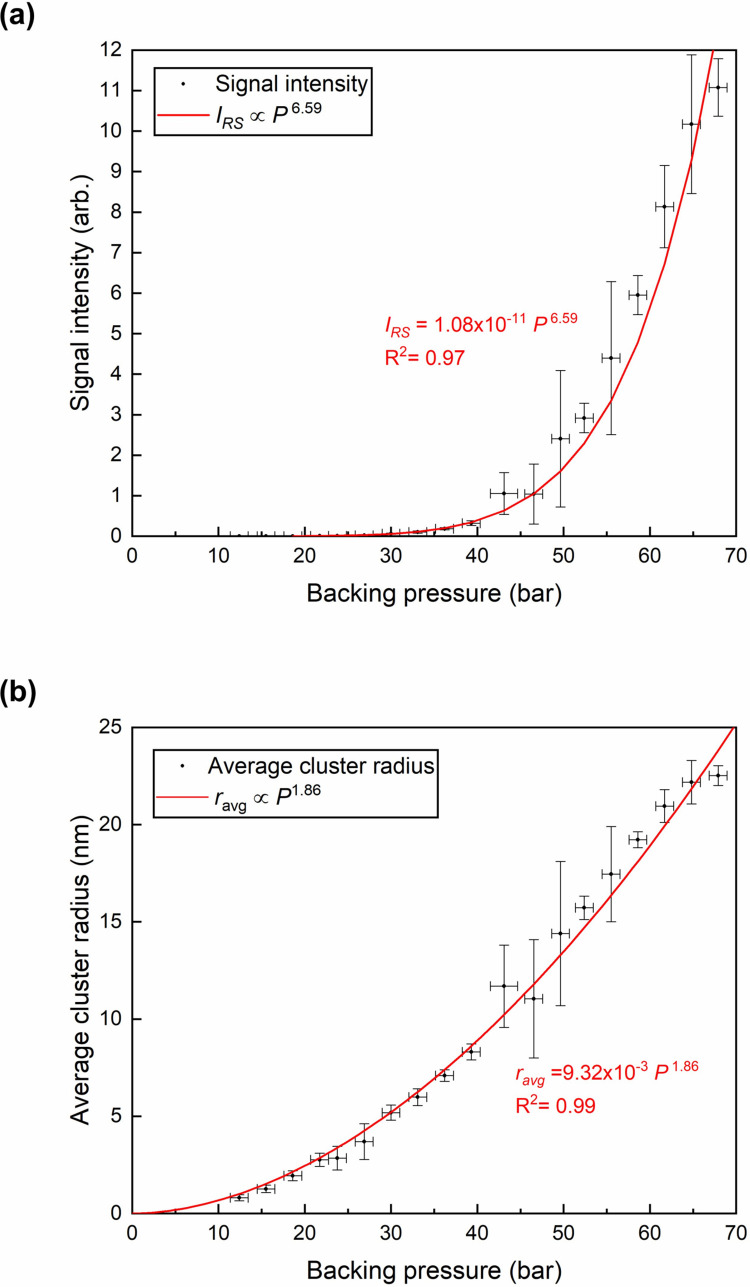
The pressure dependence of Rayleigh scattering signal intensity and the average radius of methane clusters. (a) The Rayleigh scattering signal intensity and (b) the average radius of the methane clusters with respect to the backing pressure varying from 11 bar to 69 bar. Each data point represents a 15-shot average, and error bars are obtained from their standard deviations.

Fig 3(A) shows the Rayleigh scattering intensity as a function of backing pressure. We measured the Rayleigh scattering of methane clusters at room temperature, varying the backing pressure from 11 bar to 69 bar. Each point in the figure represents a 15-shot average of the scattering signals at similar gas jet conditions, and the error bars show the standard deviations of those measurements. The methane cluster radius at the onset of clustering is found to be 6.4 Å at 11.4 bar, and the average cluster radius of methane is plotted as a function of backing pressure in Fig 3(B). The red curve in Fig 3(A) shows a power law fit to the scattering signal, which shows that the Rayleigh scattering signal intensity increases with the backing pressure as *I*_RS_∝*P*_0_^6.59^. Since the average cluster radius is proportional to ${\left(\frac{I_{RS}}{P_{0}}\right)}^{\frac{1}{3}}$ in Eq (6), we expect the radius to be proportional to *P*_0_^1.86^. The corresponding power law fit is shown as a red curve in Fig 3(B).

**Fig 4:**
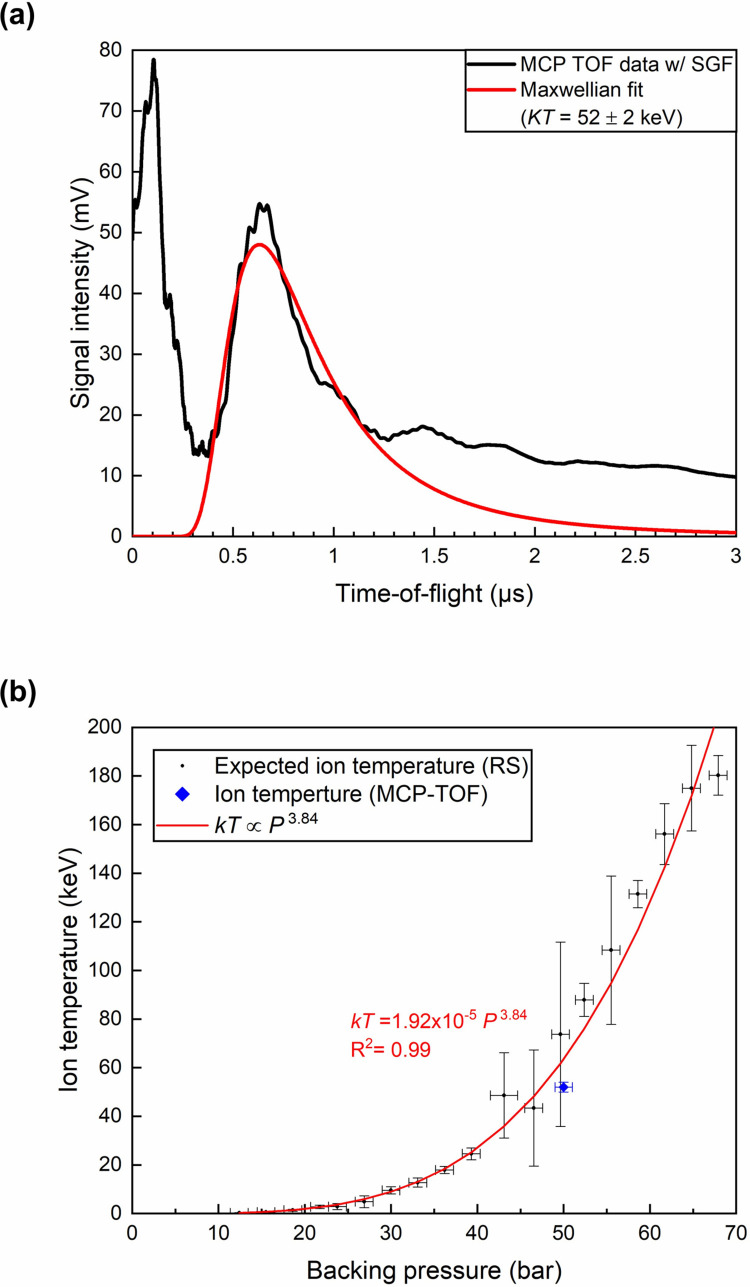
Ion time-of-flight signal and the expected ion temperature. (a) Ion time-of-flight signal from the MCP detector smoothed out with a Savitzky-Golay filter (SGF). The red curve shows a Maxwellian fit for deuterium ions with *kT* = 52 keV. (b) Expected ion temperature derived from average cluster radius with respect to the backing pressure. The blue diamond indicates the deuterium ion temperature (52±2 keV) measured from the MCP time-of-flight detector.

Previous studies report on the log-normal size distribution of clusters [22], which explains the observed Maxwellian velocity distribution of energetic ions after the Coulomb explosion. Fig 4(A) shows an example of the ion time-of-flight signal obtained from our MCP by irradiating CD_4_ clusters with an intense laser pulse (<10^18^ W/cm^2^). The backing pressure was 50 bar for this measurement. A huge initial x-ray peak is followed by the energetic deuterium ion signal. The red curve represents a Maxwellian fit for deuterium ions with *kT* = 52 keV. The residual at the tail of the ion signal is attributable to carbon ions that arrive much later than the deuterium ions. Since the incident laser intensity is below 10^18^ W/cm^2^ in our experiment, we estimate the charge state of carbon ions as 4+ [23]. Since the speed of ions after Coulomb explosion increases with the charge-to-mass ratio, we expect deuterium ions to move faster than carbon ions.

As in previous studies, we approximate the ion temperature as two thirds of the average kinetic energy of ions [8]. Fig 4(B) shows the expected deuterium ion temperatures for different backing pressures along with the actual temperature measurement at a backing pressure of 50 bar. The measured ion temperature from the MCP time-of-flight signal is shown as a blue diamond at 52 keV, which agrees with the expected temperature from methane clusters at 50 bar with *r* = 14 nm. Note that the Hagena scaling law predicts a much bigger methane cluster for our experimental condition, and the ion temperature predicted using the Hagena parameter is far off the experimentally measured ion temperature of *kT* = 52±2 keV. This is consistent with previous studies [24–26], where the Hagena scaling law overestimates the cluster sizes in their experiments.

## Conclusion

The optimum timing for the laser-cluster interaction appears to be about 800 μs after the valve opening, when the time-averaged Rayleigh scattering signal reaches its maximum. We have measured the average size of methane clusters for backing pressures ranging from 11–69 bar. Our Rayleigh scattering measurements indicate that the onset of clustering occurs at ~11 bar, where the methane cluster size is estimated as *N*_c0_≅20. The average cluster radius *r* follows the power law of *r*∝*P*_0_^1.86^. Based on these measurements, we calculate the expected deuterium ion temperatures for different gas jet backing pressures. We find that the experimentally measured deuterium ion temperature of 52±2 keV at 50 bar is consistent with these predictions, validating our cluster size determination using time-resolved Rayleigh scattering measurements of methane clusters.

## Supporting information

S1 File Detailed analysis of the time-resolved scattering measurements. (DOCX)

S1 Dataset Minimal underlying data set. (PDF)
