# Supplementary material for: Time-resolved Rayleigh scattering measurements of methane clusters for laser-cluster fusion experiments
Source: PLoS One. 2021 Dec 17;16(12):e0261574. doi: 10.1371/journal.pone.0261574 (PMC8682908; doi:10.1371/journal.pone.0261574)
Supplement: S1 File — (DOCX) [file pone.0261574.s001.docx]

**S1 file.**

**Detailed analysis of the time-resolved scattering measurements**

**S1.1 Comparison between scattering signals at ~600 μs and ~800 μs**


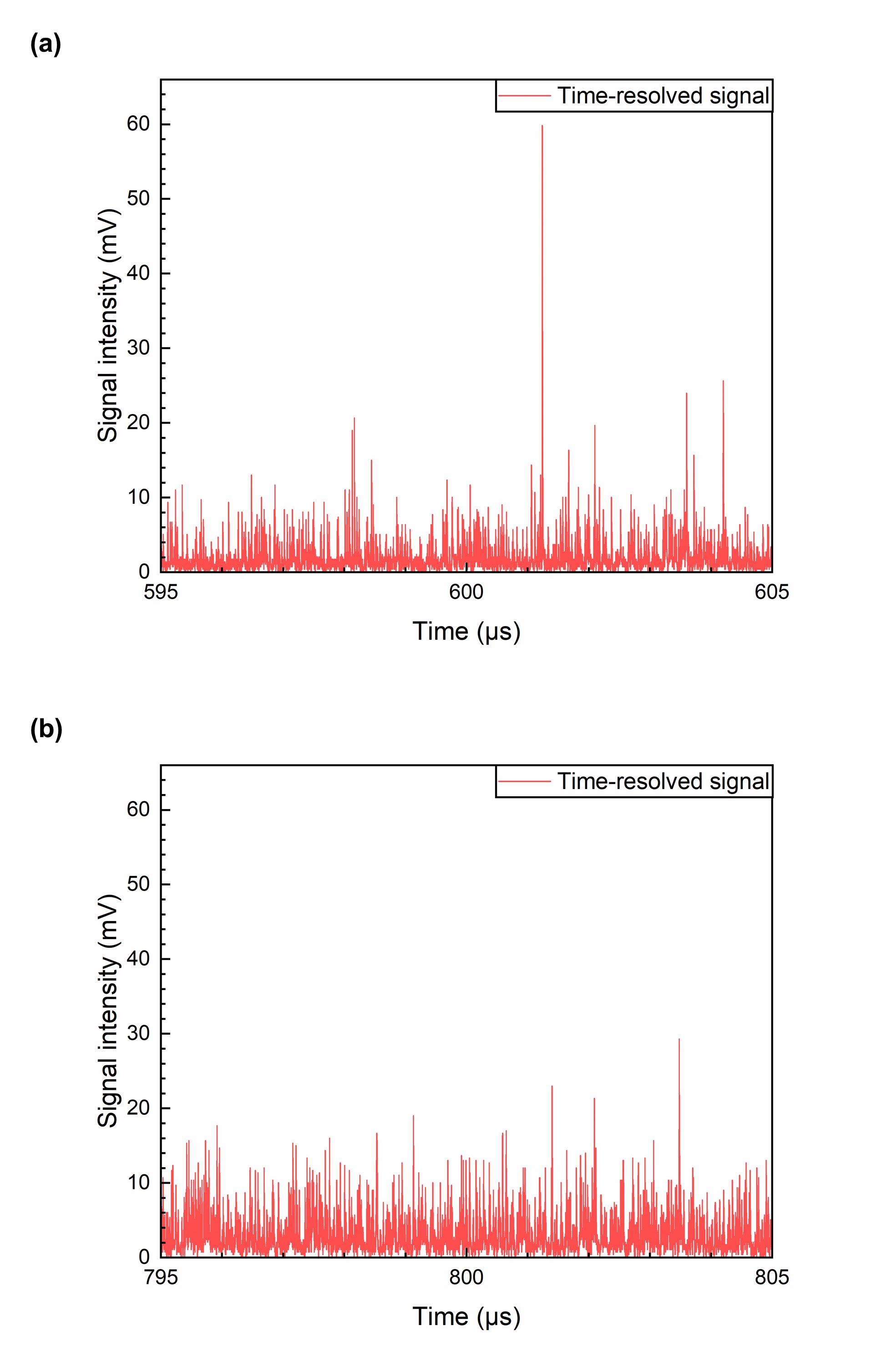


**Fig A Signals at around 600 μs and 800 μs.** The red lines indicate the time-resolved Rayleigh scattering signals for 10 μs at around (a) 600 μs and (b) 800 μs after the nozzle starts opening. The backing pressure is 20 bar.

In Fig 2, we find that the time-averaged signal (black bar) reaches its maximum at around 800 μs while several large time-resolved signals (red lines) appear at around 600 μs. Figure A (a) and (b) show magnified time-resolved signals at ~600 μs and ~800 μs, respectively, in 10 μs time windows. There is a large peak at ~601 μs in Fig A(a), but we find that there are more peaks in Fig A(b). In order to compare the amount of detected signals quantitatively, we have calculated the signal area for each case. This comparison reveals that the signal area is 19.3 mV∙μs for Fig A(a) and 28.9 mV∙μs for Fig A(b). Most of the signals in Fig A(a) and A(b) seem to correspond to single photon events as discussed in S1.3. Apparently, there is a probability of multiple photons being detected by the PMT within its decay time, and we think that this can explain the large peaks at around 600 μs in Fig 2.

**S1.2 Time-resolved Rayleigh scattering signal**


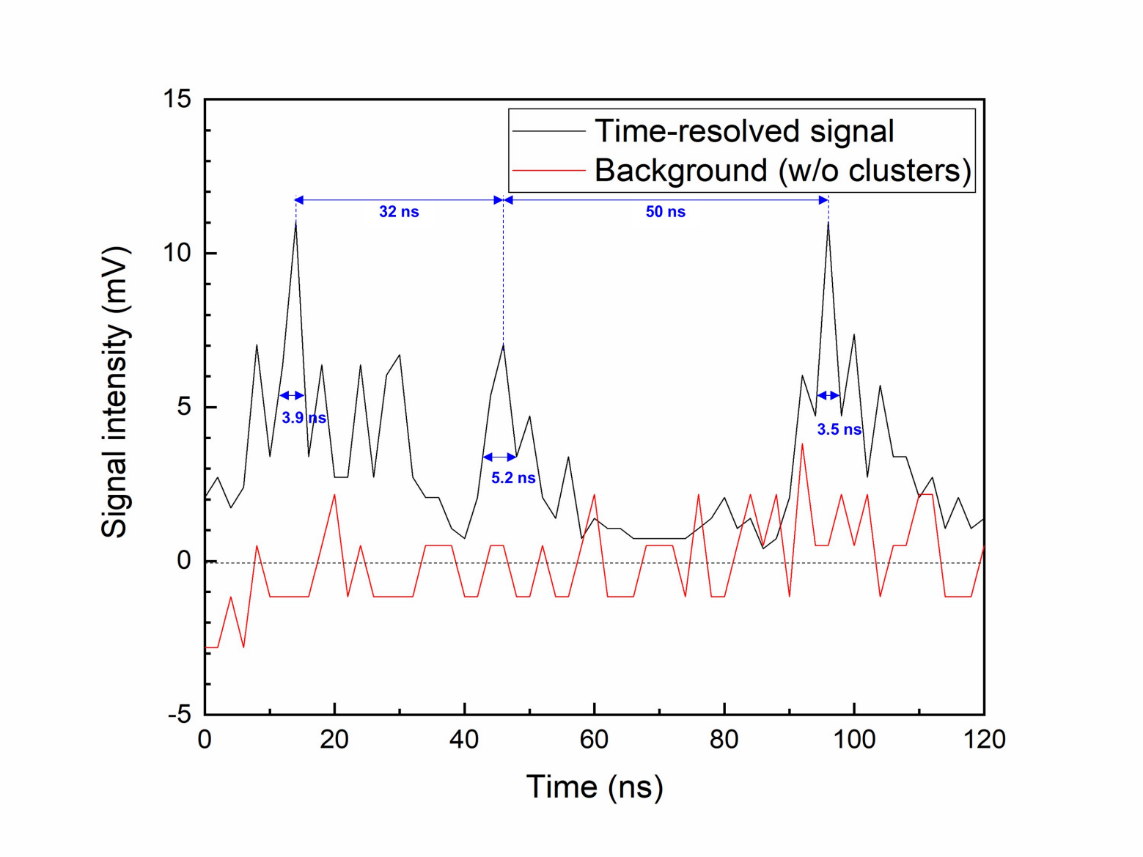


**Fig B.** **Time-resolved scattering signal in a 120 ns time window.** The solid black line shows a magnified time-resolved Rayleigh scattering signal around 800 μs in a 120 ns time window. A background noise level shown as a solid red line has been measured independently with no cluster jet, and is plotted in the same figure. The backing pressure of methane is 20 bar for these measurements.

Fig B shows an example of the time-resolved Rayleigh scattering signals around 800 μs in a 120 ns time window. The time between two adjacent peaks are 32 ns and 50 ns in this figure, which roughly agrees with our estimates detailed in the following section. Furthermore, Fig B implies that our time resolution is sufficient for single photon counting since we estimate the time resolution of our detection system to be a few nanoseconds.

The signals in our measurements seem to correspond mostly to single photon events, so we can calculate the expected signal height using the approximate pulse duration (~5 ns in Fig B) and the gain of our PMT. The signal height from a single photon detection, $V_{RS}$, is given as

$V_{RS}=\frac{e\cdot G}{\tau}\times R\approx\frac{\left( 1.6\times{10}^{-19}\left[ C \right] \right)\cdot(\sim{10}^{7})}{\sim5\times{10}^{-9} \left[ s \right]}\times50 \left[ \Omega\right]=\sim16 [mV]$,

where $e$ is the charge of an electron, *G*~10^7^ is the typical gain of R928 PMT at 1000 V, $\tau$~5 ns is the pulse duration, and *R*=50 Ω is the impedance of the oscilloscope.

**S1.3 Average time between Rayleigh scattering signals**

In this section, we attempt to estimate the average time between two consecutive scattering signals detected in our PMT. We calculate the average time between measured Rayleigh scattering signals, $T_{s}$,using the number of incident photons on the cluster jet during the 1 ms valve opening time and the scattering probability. We have used a laser line filter with a 71% transmission efficiency at 632.8 nm and a 2-inch AR coated collection lens before the PMT, and have considered those factors in our calculations.

Since we have used a 21 mW He-Ne laser as our light source, the number of photons incident on the methane cluster jet during the 1 ms opening time, *N*, can be approximated as

$$N=\frac{21 \left[ \mathrm{mW} \right]\times1 [ms]}{\frac{hc}{\left( 632.8 \left[ \mathrm{nm} \right] \right)}}=6.69\times{10}^{13},$$

where *h* is Plank’s constant, and *c* is the speed of light.

Figure 3(b) shows that we have produced methane clusters with an average radius of 2.45 nm at a backing pressure of 20 bar, which corresponds to *N_c_*=1148 molecules per cluster. Assuming a molecular number density of $n_{{CH}_{4}}$~10^17^ molecules/cm^3^ for methane cluster jet, the number density of methane clusters, $n_{c}$, becomes

$n_{c}=\frac{n_{{CH}_{4}}}{N_{c}}\approx\frac{{\sim10}^{17} \left[ \frac{\mathrm{molecules}}{\mathrm{cm}^{3}} \right]}{1148 \left[ \mathrm{molecules} \right]}=8.71\times{10}^{13} [\mathrm{clusters}/\mathrm{cm}^{3}]$.

Using Eq. (1) and *r*=2.45 nm in the formula, we calculate the differential cross-section, $\frac{d\sigma}{d\Omega}$. Accounting for a 2-inch collection lens at 120 mm from the nozzle, we approximate the effective scattering cross-section, $\sigma_{RS}$, as

$$\sigma_{RS}\approx\frac{d\sigma}{d\Omega}\Delta\Omega=\frac{16\pi^{4}\left( 2.45 \left[ \mathrm{nm} \right] \right)^{6}}{\left( 632.8 \left[ \mathrm{nm} \right] \right)^{4}}\left( \frac{\left( 1.27 \right)^{2}-1}{\left( 1.27 \right)^{2}+2} \right)^{2}\times\left( \frac{\left( CA\cdot25.4 \left[ \mathrm{mm} \right] \right)^{2}\cdot\pi}{\left( 120 \left[ \mathrm{mm} \right] \right)^{2}} \right)$$

$$=7.68\times{10}^{-23} \left[ \mathrm{cm}^{2} \right],$$

where, $\Delta\Omega$ is the solid angle covered by the 2-inch collection lens with a clear aperture (CA) of 95%.

Therefore, the number of Rayleigh scattered photons arriving at the lens during the 1 ms opening time, $N_{RS}$, becomes

$$N_{RS}=N\cdot{(n}_{c}\sigma_{RS}l)=2.24\times{10}^{5}$$

where *l*=0.5 cm is used for the scattering path length based on the nozzle exit diameter of 0.5 cm.

Accounting for the transmission efficiency of 71% for the laser line filter, and a quantum efficiency of 8% for R928 PMT, we estimate the total number of pulses measured with our PMT during 1 ms opening to be $N_{RS}\times(71\%)\times(8\%)=1.27\times{10}^{4}$ pulses.

In summary, we expect the average time between scattering signals to be *T_s_*=(1 ms)/(1.27×10^4^ pulses) ~ 78.6 ns. This value is roughly consistent with those seen in Fig B.
