# Supplementary material for: Time-resolved Rayleigh scattering measurements of methane clusters for laser-cluster fusion experiments
Source: PLoS One. 2021 Dec 17;16(12):e0261574. doi: 10.1371/journal.pone.0261574 (PMC8682908; doi:10.1371/journal.pone.0261574)
Supplement: S1 Dataset — (PDF) [file pone.0261574.s002.pdf]

P[bar]	I_RS[arb.]	xEr	yEr
12.411	0	1.034	0
15.513	0	1.034	0
18.616	0.002	1.034	0.001
21.718	0.007	1.034	0.003
23.787	0.008	1.034	0.005
26.889	0.022	1.034	0.017
29.992	0.061	1.034	0.015
33.095	0.103	1.034	0.026
36.197	0.186	1.034	0.029
39.3	0.324	1.034	0.057
43.092	1.055	1.58	0.519
46.539	1.039	1.034	0.74
49.642	2.406	1.034	1.683
52.4	2.918	1.034	0.364
55.503	4.395	1.034	1.888
58.605	5.955	1.034	0.483
61.708	8.136	1.034	1.017
64.81	10.171	1.034	1.713
67.913	11.077	1.034	0.709

P[bar]	r[nm]	xEr	yEr
12.411	0.808	1.034	0.154
15.513	1.269	1.034	0.188
18.616	1.94	1.034	0.258
21.718	2.761	1.034	0.341
23.787	2.849	1.034	0.611
26.889	3.697	1.034	0.923
29.992	5.186	1.034	0.391
33.095	5.985	1.034	0.436
36.197	7.093	1.034	0.298
39.3	8.309	1.034	0.412
43.092	11.688	1.58	2.112
46.539	11.046	1.034	3.041
49.642	14.398	1.034	3.705
52.4	15.721	1.034	0.604
55.503	17.453	1.034	2.455
58.605	19.224	1.034	0.409
61.708	20.953	1.034	0.84
64.81	22.183	1.034	1.117
67.913	22.514	1.034	0.51

P[bar]	kT[keV]	xEr	yEr
12.411	0.232	1.034	0.089
15.513	0.573	1.034	0.17
18.616	1.338	1.034	0.356
21.718	2.711	1.034	0.67
23.787	2.887	1.034	1.238
26.889	4.861	1.034	2.427
29.992	9.565	1.034	1.442
33.095	12.739	1.034	1.856
36.197	17.892	1.034	1.503
39.3	24.553	1.034	2.435
43.092	48.583	1.58	17.558
46.539	43.393	1.034	23.892
49.642	73.725	1.034	37.943
52.4	87.896	1.034	6.754
55.503	108.33	1.034	30.476
58.605	131.43	1.034	5.592
61.708	156.135	1.034	12.519
64.81	175.004	1.034	17.624
67.913	180.266	1.034	8.167
